# Introduction of a Chemical-Free Metal PDMS Thermal Bonding for Fabrication of Flexible Electrode by Metal Transfer onto PDMS

**DOI:** 10.3390/mi8090280

**Published:** 2017-09-15

**Authors:** Domin Koh, Anyang Wang, Phil Schneider, Brett Bosinski, Kwang W. Oh

**Affiliations:** SMALL (Sensors and MicroActuators Learning Lab), Department of Electrical Engineering, State University of New York at Buffalo (SUNY-Buffalo), Buffalo, NY 14260, USA; dominkoh@buffalo.edu (D.K.); anyangwa@buffalo.edu (A.W.); philschn@buffalo.edu (P.S.); brettbos@buffalo.edu (B.B.)

**Keywords:** chemical-free PDMS-metal bonding, metal and PDMS bonding, various metal transfer, flexible electrodes

## Abstract

Polydimethylsiloxane (PDMS) is a flexible and biocompatible material widely used in the fabrication of microfluidic devices, and is often studied for the fabrication of flexible electrodes. The most popular method of fabricating a flexible electrode using PDMS is done by transferring a metal electrode onto said PDMS. However, the transfer process is difficult and the transferred metal layer is easily damaged due to inherently weak adhesion forces between the metal and PDMS, thus requiring a chemical treatment or sacrificial layer between the two. The fabrication process using a chemical treatment or sacrificial layer is complicated and expensive, which is the major limitation of using PDMS in the fabrication of flexible electrodes. This paper discusses the findings of a possible solution to create strong bonding between PDMS and various metals (copper, nickel and silver) using a chemical-free metal to PDMS thermal bonding technique. This method is the same as the PDMS curing process, but with a variation in the curing condition. The condition required to create strong bonding was studied by observing copper transferred by various PDMS curing conditions, including the standard condition. The condition creating the strong bonding was baking PDMS (5:1 = base polymer: curing agent) at 150 °C for 20 min. Experimentation showed that the optimum thickness of the transferred metal shows that the optimum thickness is approximately 500 nm, which allows for a higher resistance to stresses. The successful transfer of copper, nickel and silver layers onto PDMS with a stronger adhesion force opens up many new applications dealing with the fabrication of flexible electrodes, sensors, and flexible soft magnets.

## 1. Introduction

Polydimethylsiloxane (PDMS) is a widely used polymer material in the fabrication of microfluidic devices because it is an inexpensive and biocompatible material with a high gas permeability, chemical stability, and transparency [1]. PDMS is also studied in the fabrication of flexible electrodes by deposition of a metal layer or transferring a metal electrode onto PDMS. However, due to the weak adhesion forces between PDMS and metal, neither the deposition nor transfer of a metal electrode onto PDMS is easy. Therefore, a complicated process is required to enhance the adhesion between metal and PDMS using a chemical treatment or sacrificial layers [2,3,4,5,6,7,8,9,10]. For example, Byun et al. successfully transferred gold electrodes on PDMS by treating gold electrode with (3-mercaptopropyl)trimethoxysilane (MPTMS) for 20–540 min [5,6]. Chemical treatment is a time-consuming process that results in longer and complicated fabrication processes. As a result, the fabrication of flexible electrodes by printing conductive ink has recently been considered a better method [11] due to the fact that it is simpler (excluding preparation of ink). The limitation of printing is that the preparation of conductive ink is very complex and requires expensive equipment. However, the use of chemical treatments in the process of metal transferring onto PDMS can be as complicated as the process of preparing the conductive ink.

Recently, we had discovered a simple method to create strong adhesion between PDMS and various metals. This was done without chemical treatments and sacrificial layers through metal PDMS thermal bonding. This method is the same as the PDMS curing process (using uncured PDMS), but the curing conditions (such as PDMS mixing ratio, baking temperature and baking time) are modified to promote stronger adhesion. The fabrication of the electrode still requires conventional methods such as photolithography and etching; however, it avoids the use of chemical treatments in transferring the electrode onto PDMS. The proposed method is simpler and less expensive, as it does not require any chemical treatment, and the creation of the strong adhesion is completed in the PDMS curing process. The simplicity and low cost fabrication are not the only advantages of our method. For instance, it is compatible with various metals, thus it can be used in the fabrication of various devices such as flexible electrodes, flexible soft magnets, and microfluidic devices with integrated soft magnetic structures (for bioassay or particle sorting). This would be possible by using the idea of fabricating a ceiling substrate and PDMS microfluidic channel wall structure separately (for fabricating glass-PDMS-glass configuration) [12,13]. The metal structure transferred PDMS can be either a ceiling substrate or microfluidic channel wall structure.

In this paper, we demonstrated the fabrication of a flexible copper electrode as a simple application of our method by transferring the copper electrode onto PDMS to demonstrate the chemical-free metal PDMS thermal bonding. Firstly, the conditions that create strong bonding between copper and PDMS was studied by observing the copper layer transfer onto PDMS. As a simple proof of concept that our method enhances the bonding strength between the metal and PDMS, the adhesion strength generated by our method was compared to that of standard PDMS curing conditions. Then, the change in the sheet resistance (before and after transfer) of different thicknesses of the copper electrode was studied to find the optimum thickness in terms of its resistance to stress generated during the fabrication process. To further demonstrate the application, we fabricated and tested a capacitive pressure sensor. This offers greater flexibility and versatility of the electrode. Additionally, we studied the stability of the electrode by measuring the resistance change while bending the electrode to create deformation. Lastly, the successful enhanced transfer of other metals (nickel and silver) onto PDMS was demonstrated.

## 2. Method/Experiment

### 2.1. Demonstration of Metal Transfer and the Study of Various PDMS Curing Conditions

**Figure 1:**
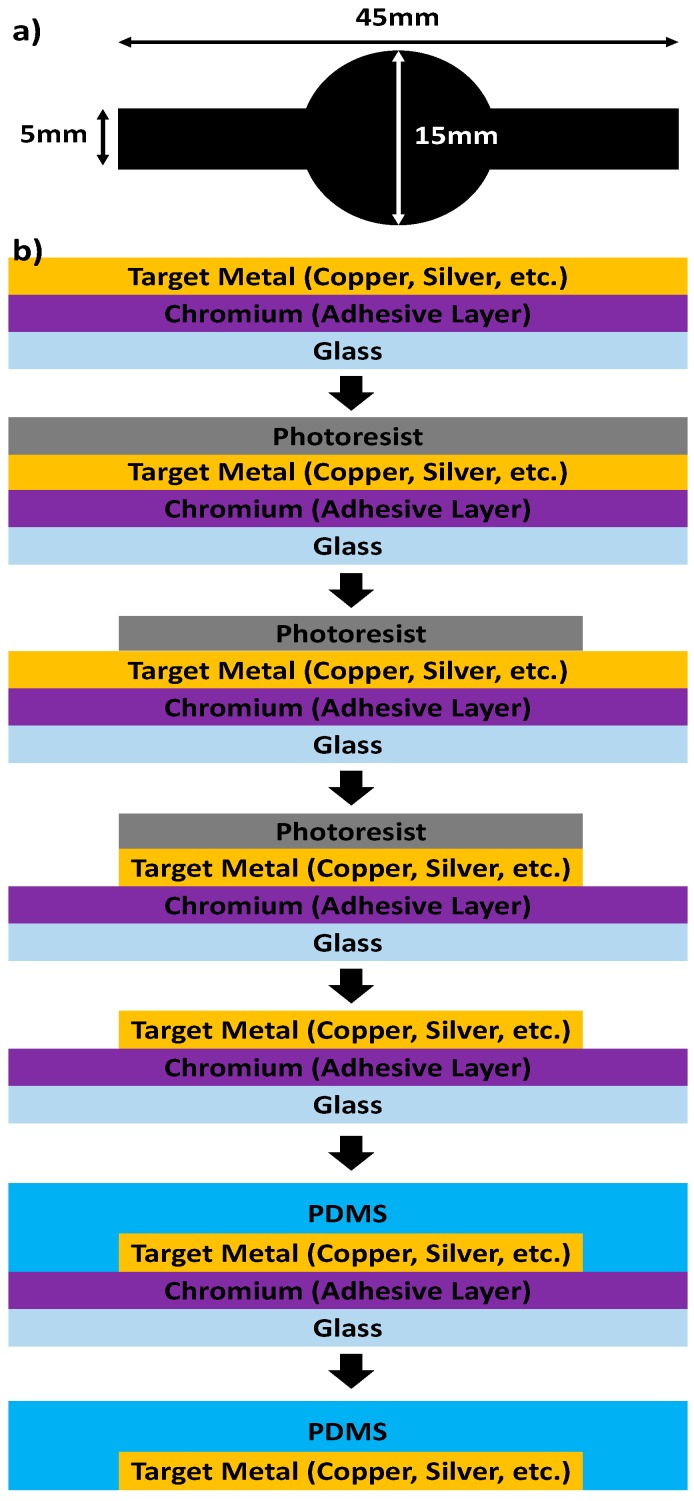
An illustration of chemical-free metal polydimethylsiloxane (PDMS) thermal bonding method. (**a**) A simple design for a mold to create a copper layer pattern. The mold was composed of a rectangular pattern size of 5 mm × 45 mm and a large circle at the center (diameter is 15 mm); (**b**) illustrations of the metal transfer process. Initially, metal layers were deposited on glass, firstly chromium (adhesive layer) then the target metal such as copper. The pattern of the target metal was fabricated by conventional photolithography and wet etching. Uncured PDMS was then poured on the metal surface. The curing conditions such as the PDMS mixing ratio, baking temperature and baking time was varied based on optimal adhesion parameters. After the PDMS was cured, the PDMS was peeled off of the transferred target metal layer.

The process of chemical-free, metal PDMS thermal bonding for transferring electrodes onto PDMS is shown in Figure 1. Initially, a 10 nm thick chromium layer was deposited as an adhesive layer on a glass substrate. Next, the target metal, copper for this demonstration, was deposited by an Electron-beam metal deposition system (Kurt J. Lesker Company AXXIS, Jefferson Hills, PA, USA). The purity of metal source is 99.999% and there was no vacuum broken between chromium and copper deposition. The pressure was set to 1 × 10^−6^ Torr and the deposition rate was set to 0.5 Å/s. The thickness can be varied to any thickness depending on its purpose. For a simple observation of metal transfer, we designed a large pattern mask as shown in Figure 1a. The designed mask was composed of a rectangular pattern size of 5 mm × 45 mm and a circle at the center (diameter of 15 mm). A 10 µm thick SU-8 (SU-8 2010, Micro-Chem Corp., Newton, MA, USA) photoresist layer was deposited on the copper, and then exposed to UV light through the mask. After developing the photoresist, the copper was etched using Ferric Chloride, (MG Chemicals, Burlington, ON, Canada), and the photoresist layer was removed by acetone bathing. Finally, uncured PDMS (Sylgard 184, Dow Corning Corp., Midland, MI, USA) of different mixing ratios was poured on the surface of copper, and then baked at different baking conditions.

**Figure 2:**
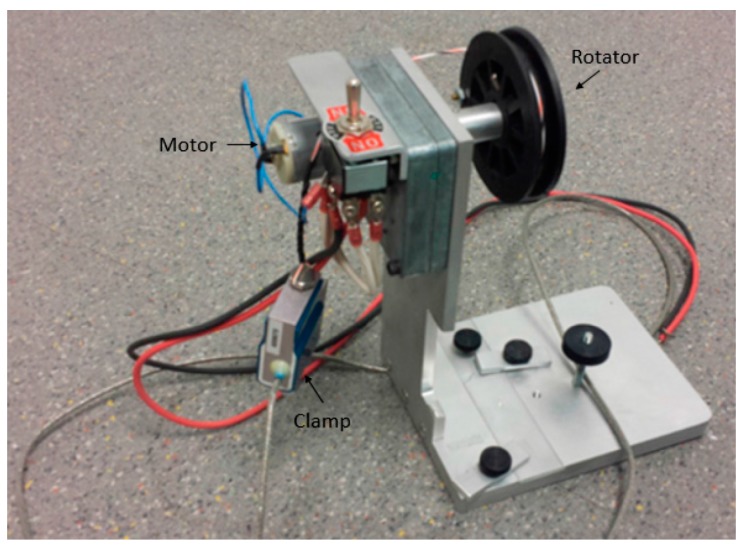
A homemade PDMS lifting equipment. After peeling an edge of the PDMS, using the clamp of the equipment, the PDMS was peeled at ~1 mm/s.

For the study of the condition creating strong bonding, we varied the baking temperature, PDMS mixing ratio, and baking time. These three parameters are the major factors of creating a strong adhesion. The PDMS was prepared at different mixing ratios: 20:1, 10:1 and 5:1 (weight ratio of base polymer: curing agent). These were then baked at different baking conditions: 100 °C for 20 min, 30 min and 45 min, and 150 °C for 20 min, 30 min and 45 min. The PDMS peeling was started from one edge; it was held 90° from the glass substrate and carefully pulled at ~1 mm/s using equipment shown in Figure 2.

The effect of vacuum was considered as a minor factor at this time since the existence of vacuum had no significant effect in creation of strong bonding. Therefore, applying the vacuum was excluded in the PDMS curing condition.

### 2.2. Bonding Strength Test

As a proof of concept, we used a cross-hatch adhesion test to study and compare the adhesion strength generated by our method and by the standard PDMS curing process [14].

We fabricated two samples of transferred copper layers onto PDMS. One sample was fabricated by our method, PDMS with a 5:1 mixing ratio was baked at 150 °C for 20 min. Another was fabricated using standard curing conditions, PDMS of 10:1 mixing ratio was baked at 95 °C for 45 min. In the copper layer transferring process using standard curing conditions, the copper layer was transferred from a glass substrate (no adhesive layer was deposited on glass substrate). Then, a series of cross cuts was made by making cuts vertically and horizontally (cuts were made every 1 mm) on the transferred copper and the surface was brushed five times. Next, we attached a tape, Elcometer 99 ASTM D3359 (Elcometer, Rochester Hills, MI, USA), which is equivalent to Permacel P99 tape, to the surface of copper and the tape was peeled rapidly at 180°. The adhesion strength between the transferred copper and PDMS can be obtained by analyzing the removed area of the copper squares according to the ASTM (American Society for Testing and Materials) classes.

### 2.3. Study of Optimum Electrode Thickness

According to the study of Lu et al. in 2009 [15], when stress is applied to the flexible electrode, the thickness of electrode affects its electrical stability. As shown in the Figure 1, the fabrication process involves deformation of the PDMS and electrode in the peeling off step. Therefore, the study of the optimum thickness that is more resistant to stress is important in order to minimize the damage to the transferred metal electrode. Here, the optimum thickness was studied by comparing the change in sheet resistance before and after the transfer of a series of different copper thicknesses.

Copper layers of different thickness (75 nm, 150 nm, 300 nm, 500 nm, and 1000 nm) were transferred onto PDMS using the process in Figure 1 and the same copper layer deposition condition. Then, the change in sheet resistance before and after transfer was measured using an Alessi CPS-7089-17 Four Point Probe which connected to a Keithley 228A (Tektronix, Inc., Beaverton, OR, USA) voltage/current source and Keithley 195A digital multimeter (Tektronix, Inc.).

### 2.4. Study of the Resistance Change Flexible Electrode

The resistance change caused by bending of the transferred copper electrode was measured using cylinders of different radii. A series of rectangular flexible copper electrodes (size: 30 mm × 7 mm) of different thicknesses (300 nm, 500 nm and 1000 nm) were fabricated by the chemical-free metal PDMS thermal bonding, which is shown in Figure 1. The change in the resistance of the flexible electrode was measured after bending it around cylinders of radii: 55 mm, 45.5 mm, 35 mm, 32 mm, 24.5 mm, 19 mm, 13.5 mm, 11 mm, and 5 mm. The resistance was measured five times and the average was calculated with errors.

Moreover, the flexible electrodes were bent by a cylinder of 11 mm radius repeatedly for 1000 bending cycles to measure the resistance change to study the stability of the electrodes against repetitive deformation.

### 2.5. Study of Pressure Sensor

**Figure 3:**
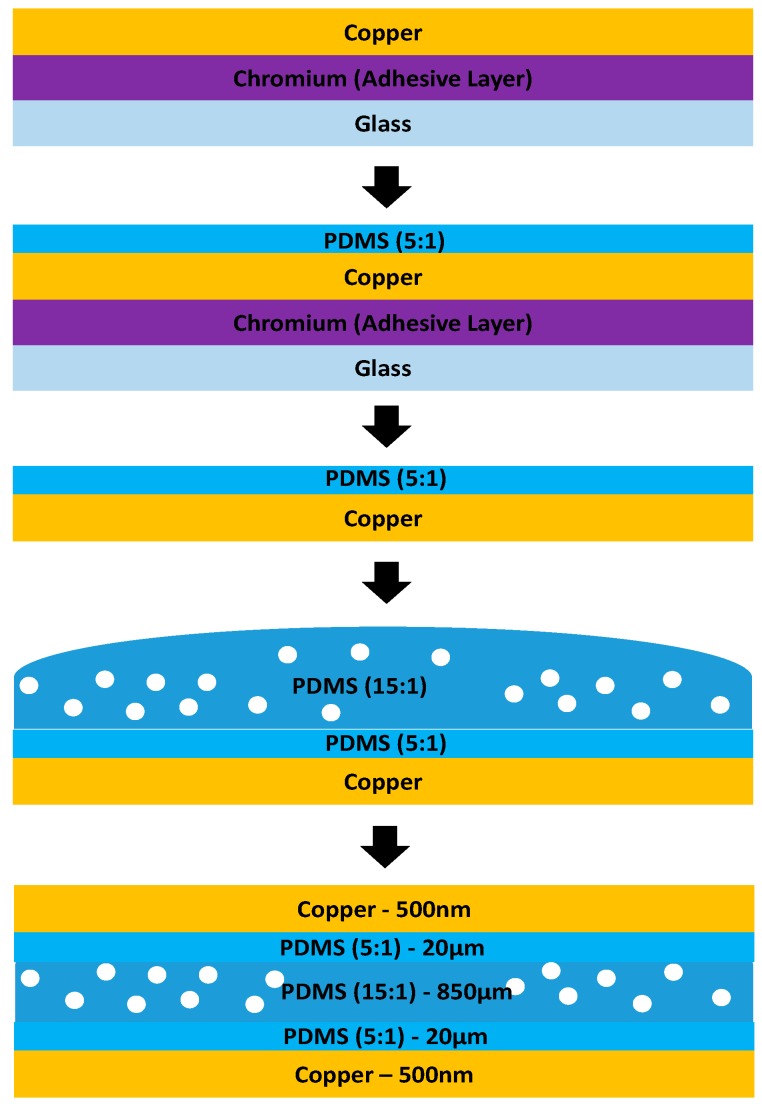
Illustration of the fabrication process of pressure sensitive capacitor. Initially, a 10 nm thick chromium (adhesive layer) layer was deposited and then a 500 nm thick copper layer was deposited. The copper layer was transferred onto 20 µm thick PDMS by baking it at 150 °C for 20 min. Two transferred copper electrodes were prepared and sandwiched PDMS (mixing ratio of 15:1) with water mixed (4:1 = PDMS:water in the weight ratio). Two transferred copper electrodes were spaced by 850 µm thick spacer then baked at 95 °C for 45 min.

As a further demonstration of our method, we fabricated a capacitive pressure sensor, which is sensitive to a wide range of pressure by the process illustrated in Figure 3. Initially, two transferred copper electrodes were fabricated by transferring 500 nm thick copper onto PDMS (5:1 mixing ratio). After deposition of a 10 nm thick chromium layer and a 500 nm thick copper layer on a glass substrate, the 20 µm thick PDMS was spun coated on the copper then baked was baked at 150 °C for 20 min then peeled off. Next, we prepared a liquid PDMS (mixing ratio 15:1) mixed with DI water (4:1 = PDMS: DI water in weight ratio) [16] and poured between the flexible electrodes. We sandwiched two electrodes to create a dielectric layer between two electrodes by placing four 850 µm thick spacers at each corner and baked it at 95 °C for 45 min. After curing the PDMS, a rectangular capacitive pressure sensor, size of 55 mm × 21 mm × 890 µm, was obtained by removing edges using a knife.

The composition of dielectric layer would be sensitive to different ranges of pressure. In order to study this, we applied pressure from 1 Pa (touching) to 131.83 kPa and measured the capacitance change. The change in capacitance was measured by an Impedance analyzer 4294A (Keysight Technologies, Santa Rosa, CA, USA).

### 2.6. Study of Different Metal Transfers

The chemical-free metal PDMS thermal bonding can create strong bonding between PDMS and various metals. Aside from copper, nickel and silver were studied for the demonstration of transferring other metals. We designed a mask of micro-size line patterns (400 µm, 300 µm, 200 µm and 100 µm) for fabricating the micro-size line patterns of nickel and silver. This is a demonstration of transferring nickel and silver as well as transferring a micro-size pattern onto PDMS.

On the glass substrate, there was 10 nm thick chromium and a photoresist, S1813 (Micro-Chem Corp., Newton, MA, USA). A mold was made using photolithography and 150 nm thick nickel or silver was deposited (using the same deposition conditions as the copper deposition); then the photoresist was removed by using an acetone bath. After this, PDMS of 5:1 mixing ratio was cured on the metal surface by baking at 150 °C for 20 min and then peeled off.

Moreover, we measured the adhesion between PDMS and nickel/silver by the cross-hatch adhesion test using the same procedure described in copper adhesion test. After making the cross-cuts, we attached an Elcometer 99 ASTM D3359 tape and then peeled it at 180° rapidly. The ASTM class can be determined by observing the percentage of area removed by tapes.

## 3. Results and Discussion

### 3.1. Results of Metal Transfer Condition

The formation of a strong bonding between PDMS and copper can be observed as a transferred copper layer on PDMS. The copper layer was transferred from the chromium layer, which acts as an adhesive layer between the glass substrate and a copper layer for easier handling during photolithography and wet etching. Without an adhesive layer, the target metal can be easily damaged. Moreover, it is evidence to show that our method creates strong bonding between the metal and PDMS.

**Figure 4:**
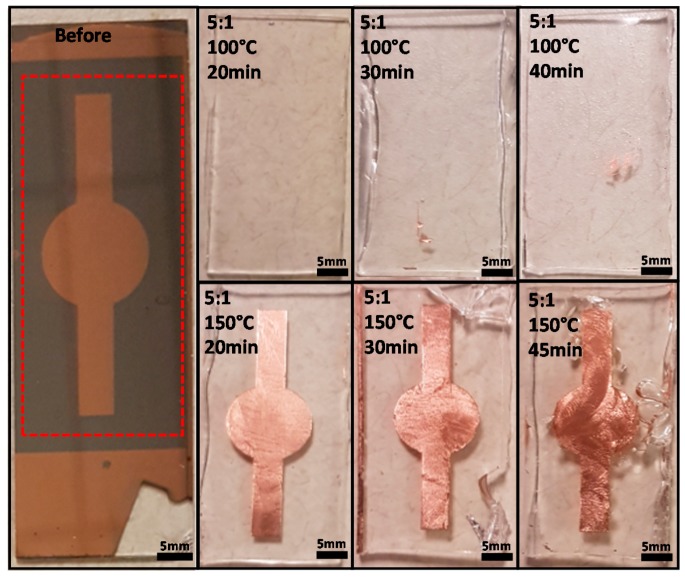
The images of the transferred copper layer on PDMS using different PDMS curing conditions. The copper layer like shown in the image ‘before’ (we only focused the pattern in dash box) was transferred from the copper layer onto PDMS. Although the PDMS was baked at a high temperature for a long time, no copper layer was transferred in the case of using the PDMS of 20:1 and 10:1 mixing ratios (excluded from the images). In the case of using the PDMS of a 5:1 mixing ratio, the transferred copper was observed on PDMS, but, if the temperature was 100 °C, the copper is partially transferred.

The theory of our method in transferring a metal layer onto PDMS is creating stronger adhesion between the target metal (copper) and PDMS than adhesion between the target metal and adhesive layer. If the adhesion between PDMS and the copper layer is stronger than the adhesion between the adhesive layer and the copper layer, the copper layer can be transferred onto PDMS. The standard PDMS curing condition cannot generate strong bonding between any metal and PDMS, and, as a result, the standard condition cannot transfer the copper layer onto PDMS. The standard condition of curing PDMS is baking a PDMS of 10:1 mixing ratio at 65–95 °C for 15–120 min. We modified the PDMS curing conditions to create a strong bonding between PDMS and the copper layer by PDMS curing process as shown in Figure 4 and Table 1.

In the case of using the PDMS of 20:1 and 10:1 mixing ratio, no copper transfer was observed even though the baking temperature was high and the baking time was long. For PDMS of a 5:1 mixing ratio, the transferred copper layer was observed. The results showed that it was possible to transfer copper with a low baking temperature (100 °C). However, in the case of the low baking temperature, the baking time must be longer than 20 min for initiating the copper transfer and only a small amount of the transferred copper was observed (partial transfer). The percentage area of transferred copper was analyzed using ImageJ software (NIH, Bethesda, MA, USA) by comparing the area of transferred copper and the total area of the copper pattern. When the PDMS was baked for 30 min, ~4% of the copper layer was transferred. When the PDMS was baked for 45 min, about 6% of the copper layer was transferred. This shows that the critical conditions for generating a strong adhesion between copper and PDMS are a greater amount of curing agent mixed in the PDMS mixture (5:1 mixing ratio), high baking temperature (150 °C) and longer baking time. Additionally, when the PDMS of 5:1 mixing ratio was baked at 150 °C for more than 20 min, the PDMS started to bond with chromium and PDMS was broken while peeling off. The chromium layer is initially deposited on a glass substrate prior to copper deposition. After copper etching, parts of the chromium layer are exposed to air (or PDMS in the PDMS curing process). This indicates that it is possible to create bonding selectively by controlling baking time.

This was a simple comparison between standard PDMS curing conditions and our modified curing condition in the creation of strong bonding. As the result shows, the condition required for generating strong bonding between copper and PDMS is a baking PDMS mixing ratio of 5:1 at 150 °C for 20 min. In addition, we expect that our method is compatible with various metals by modifying the PDMS curing conditions such as increasing baking time.

The science of creating strong bonding by changing PDMS curing conditions is not yet fully understood. It requires an extensive amount of study, especially related to surface modification caused by our PDMS curing condition.

### 3.2. Bonding Strength Test Results

**Figure 5:**
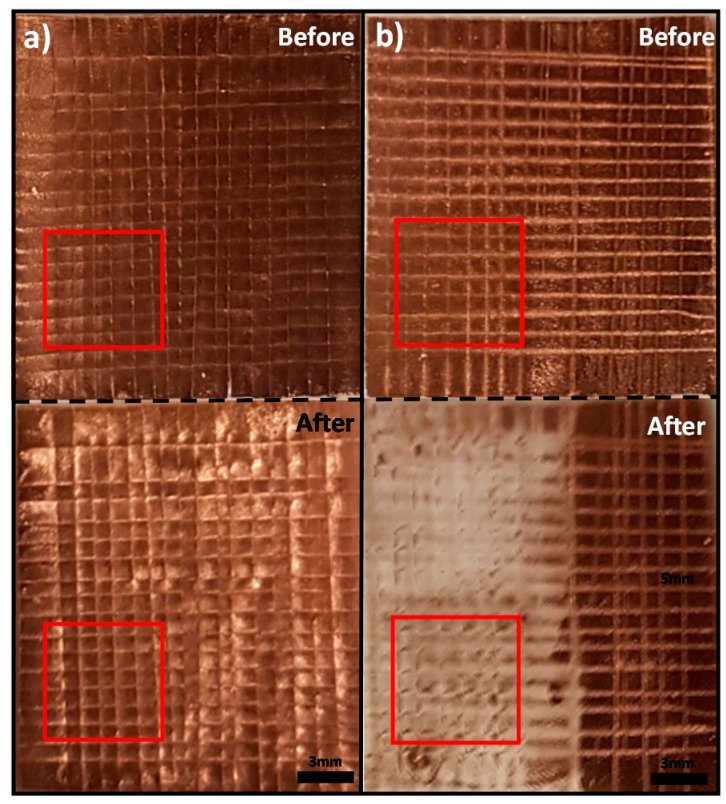
The image of the copper peeled by the different tapes after a cross-hatch adhesive test. The red square is where we analyzed the peeling of the copper squares. (**a**) The copper was transferred by our proposed method. No copper was peeled with the tape and this indicates that the ASTM class of adhesion strength of the transferred copper using our modified PDMS curing condition is 5B; (**b**) the copper layer was transferred from a glass substrate by the standard curing condition. The transferred copper layer was easily removed by the ASTM D3359 tape. This indicates that the ASTM class of adhesion strength of the transferred copper using our modified PDMS curing condition is 0B.

The cross-hatch adhesion test was repeated five times and the results are shown in Figure 5. According to the ASTM adhesion strength class, the cross-hatch test is divided into six classes, 0B–5B. 5B indicates that the removed area is 0% and 0B indicates that the removed area is greater than 65%. Due to the flexible nature of the PDMS, the distance between some cuts was not uniform. Therefore, we analyzed the area in the red box where the cuts are uniform. As shown in the Figure 5a, no copper was removed by the tape (0.1% ± 0.03%, area analyzed by ImageJ software). This indicates that the ASTM class of copper transferred by our modified method is 5B. In the case of the transferred copper by standard condition, more than 65% area was removed by the tape (82% ± 4%, area analyzed by ImageJ software), which means that the ASTM class is 0B. This proves that our method had successfully improved the adhesion strength between a copper layer and PDMS.

If the copper layer is deposited on a glass substrate without an adhesive layer, it is possible to transfer copper onto PDMS by the standard curing condition without any chemical treatment. However, this will result in a weak adhesion between copper and glass substrate, which, in turn, can be peeled off with great ease [17]. This is why the adhesion strength of transferred copper using standard condition is classified as 0B.

### 3.3. Result of Optimum Electrode Thickness

The sheet resistance of various copper thicknesses was measured 10 times sequentially and the values were averaged. As shown in Table 2, the sheet resistance was increased after the transfer, if the copper thickness was ≤150 nm, but, if the copper thickness was ≥300 nm, the sheet resistance decreased after the transfer. Based on the results, the rate of sheet resistance decreased at a copper thickness of 500 nm. Based on the experimentation, it can assumed that the optimum copper thickness is approximately 500 nm after peeling.

Initially, we expected that the sheet resistance of the electrode (of any thickness) would increase after transfer due to the cracks and wrinkles on copper surface during the PDMS peeling process. The increase rate of sheet resistance would be inversely proportional to the thickness of the electrode. However, the result showed that the sheet resistance decreases after the transfer if the thickness of copper is ≥300 nm.

**Figure 6:**
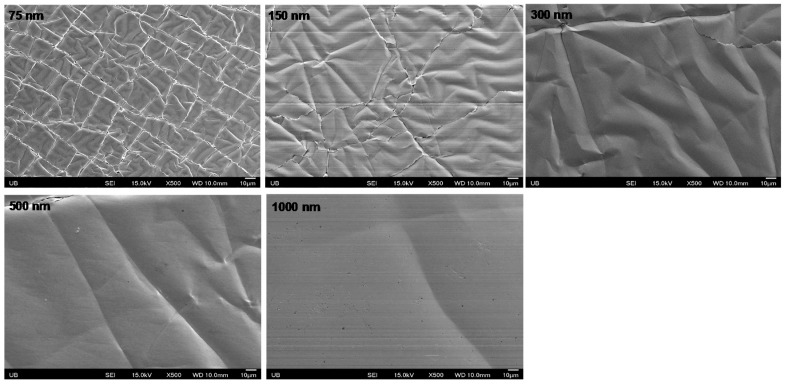
SEM images of transferred copper of different thickness, 75 nm, 150 nm, 300 nm, 500 nm and 1000 nm. The SEM images show that the thicker copper electrode had fewer wrinkles and cracks (the surface is smoother). Thus, the surface of the 75 nm thick copper electrode looks very rough due to dense wrinkle and crack, but the surface of the 1000 nm thick copper electrode looks very smooth because only a few cracks and wrinkle were observed on the surface.

To study the relationship between metal thickness and the amount of cracks and wrinkles generated on the surface, a series of SEM images of each copper thickness were taken as shown in Figure 6. The images show that more wrinkles and cracks were present on the surface of the thinner copper. The surface of the 75 nm thick copper electrode was the roughest and the surface of the 1000 nm thick copper electrode was the smoothest. If the surface smoothness caused by the creation of cracks and wrinkles is the major cause of change in sheet resistance after transfer, then the sheet resistance of 1000 nm must have the greatest decrease after transfer. However, the result in Table 2 shows that the 500 nm thick copper electrode has the greatest decrease rate of the sheet resistance after transfer. This result is similar to the study of Lu et al. in 2009 [15], which showed the effect of the copper thickness on the failure strain and that the 500 nm thick copper electrode was more resistant to the strain. In their study, they calculated the yield strength of different copper thicknesses using the Hall–Petch relation *σ_y_* = *σ*_0_ + *kd*^−1/2^ [15,18,19] where *σ_y_* is the yield strength, *σ*_0_ is the material strain constant coefficient of starting dislocation motion, k is the constant strengthening coefficient and *d* is the grain diameter. The Hall–Petch relation indicates that the yield strength of metal film is proportional to *d*^−1/2^; therefore larger grain size will lead to lower yield strength of the copper electrode. The grain size is highly dependent on various conditions such as metal deposition rate and temperature, but, according to the study of Dammers and Radelaar [20,21,22,23,24], and the grain diameter is directly proportional to square root of film thickness. Since the copper deposition condition for every thickness was identical, we can assume that the thicker copper layer would have larger grain size. In the study of Lu et al. in 2009 [15], the deposition condition for all copper thickness was identical and the grain size was increased as the thickness of copper electrode was increased. Similarly, our copper deposition condition was identical for all thickness, therefore we assume that the thicker copper electrode would have a smaller yield strength, which results in less durability of, in this case, the 1000 nm thick copper electrodes.

According to the Hall–Petch relation, a thin copper electrode (<200 nm) has a greater yield strength, which means that it can endure more stress. However, it experiences intergranular fractures as stress is applied; hence, more cracks are generated (rougher surface) and the sheet resistance is increased significantly. On the other hand, an electrode thickness above 200 nm experiences transgranular fractures from yield strength and less cracking is generated. This leads to the smoother surface of copper electrode allowing it to endure greater stresses [15].

A topic of further exploration for this research is the investigation, in terms of analytical data and explanation, of the decrease in sheet resistance of the transferred copper thickness is above 300 nm. Based off the current research, the strongest explanation for this is due to the decrease of the electrode length during the thermal expansion and compression. Applying a high temperature (150 °C) is the key of creating a strong bonding without any chemical treatment in our method, which causes thermal expansion PDMS when heated and then compression when cooled. According to the study of Bowden et al. [25,26], the PDMS is expanded by heat (thermal expansion), which creates wrinkles as it cooled down (compressed). The cracks on the copper surface suggests that there could be a thermal expansion mismatch between the copper and PDMS (additional shrinking in polymerization). The thermal expansion coefficient of copper is 17 × 10^−6^ °C^−1^ and PDMS is 3 × 10^−4^ °C^−1^ [25,26,27,28,29]. The large difference in thermal coefficient between two materials cracks and wrinkles the surface of copper after cooling down. The surface of PDMS bonded to metal would not be expanded as much as the other side; however, the thermal expansion of the surface of the PDMS bonded to the metal would be enough for the creation of wrinkles when it compressed. As a result, the length of the copper electrode was decreased, which led to the decrease of sheet resistance after transfer (*R* = *ρl/A*, where *R* is resistance, *ρ* is resistivity, *l* is the length of the electrode and *A* is the cross sectional area of the electrode). The difference in the decrease rate is caused by the fracture mechanism of the 500 nm thick copper being more resistant to stress than that of the 1000 nm thick copper [15]. Thus, the decrease rate of the 500 nm thick copper is greater than that of the 1000 nm thick copper.

### 3.4. Result of the Resistance Change Flexible Electrode

**Figure 7:**
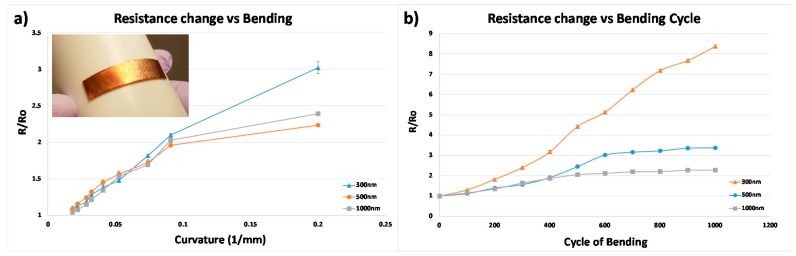
The graph shows the change in resistance as the transferred rectangular copper electrodes (30 mm × 7 mm) were bent. The change in resistance was measured in terms of the ratio (*R*/*R*_0_), *R* is the changed resistance and *R*_0_ is the initial resistance without bending. (**a**) The graph of change in the resistance as the electrodes were bent by a cylinder of different radius. The resistance of the 300 nm thick electrode was increased to 3.02 (*R*/*R*_0_). The resistance of the 500 nm thick electrode was increased to 2.24 (*R*/*R*_0_). The resistance of the 1000 nm thick electrode was increased to 2.39 (*R*/*R*_0_); (**b**) the graph of the change in the resistance as the electrode was bent repetitively by a cylinder of radius 11mm. The resistance of the 300 nm thick electrode was increased to 8.37 (*R*/*R*_0_) at 1000 bending cycles. The resistance of the 500 nm thick electrode was increased to 3.37 (*R*/*R*_0_) at 1000 bending cycles. The resistance of the 1000 nm thick electrode was increased to 2.27 (*R*/*R*_0_) at 900 and 1000 bending cycles.

The change in resistance by bending the rectangular flexible copper electrodes using the cylinders of various radii was measured and shown in Figure 7a. The resistance change was measured 10 times repetitively and then the results were averaged. The results show that the 500 nm has the least increase of the resistance. The original resistance of 300 nm, 500 nm and 1000 nm thick copper electrode were 0.88 Ω, 0.4 Ω and 0.28 Ω, respectively. The resistance change of the 300 nm, 500 nm and 1000 nm thick copper electrode after the bending using the cylinders of various radii were 3.02 (*R*/*R*_0_), 2.24 and 2.39, respectively. The result shows that the resistance increase of the 500 nm thick copper electrode was the most stable and the 300 nm thick electrode shows the greatest resistance increase. As mentioned in the study of Lu et al., the 500 nm thick electrode has the least increased rate of resistance, which is lower than the 1000 nm thick electrode because strain to rupture 500 nm is better than that of the 1000 nm due to the fracture mechanism.

The result of the change in resistance of the flexible electrode by repetitive bending using a cylinder of radius of 11 mm is shown in Figure 7b. The result shows that, as the thickness of the copper electrode increases, the rate of the resistance increase is smaller. The resistance of the 300 nm thick copper electrode after 1000 bending cycles was increased to 8.37 (*R*/*R*_0_) times greater than its original value (0.91 Ω), and it seems that it would increase after more bending cycles. The resistance of the 500 nm thick copper plate at 1000 bending cycles was increased to 3.37 (*R*/*R*_0_) times greater than its original value (0.45 Ω), though it seems that the resistance stayed constant after 900 bending cycles. The resistance of the 1000 nm thick copper plate at 900 and 1000 bending cycles was increased to 2.27 (*R*/*R*_0_) times greater than its original value (0.32 Ω). 600 bending cycles and fewer the resistance stayed constant.

The *R*/*R*_0_ value of the 500 nm thick copper electrode was lower than the 1000 nm thick copper electrode below 400 bending cycles, which follows a similar trend as the bending radii test. However, the *R*/*R*_0_ of the 500 nm thick copper electrode increased rapidly beyond 400 bending cycles, and it became higher than that of the 1000 nm thick copper electrode. This result shows that the thicker electrode is more stable against repetitive bending. The maximum stress that the electrode can endure depends on the thickness of the electrode and its grain size (which is proportional to the thickness of film). Thus, in selecting the thickness of the electrode, the optimum thickness must be selected for stability against the deformation of flexible electrode as well as choosing the right material for the electrode.

Despite the advantage of our method being simple and cheap, the defect and deformation of electrode created during the transfer process limits our method. Cracks are generated as shown in the SEM images in Figure 6 due to the mismatch of thermal expansion coefficient [25,26,27,28,29]. The crack and wrinkles cannot be avoided at this point; however, there are a few strategies to overcome this limitation. One strategy is applying the serpentine electrode design [30,31,32,33]. The serpentine design mechanically enhances the metal resistance to cracking; hence, we will design electrodes before transfer through photolithography for better quality in future studies.

### 3.5. The Result of Pressure Sensors

**Figure 8:**
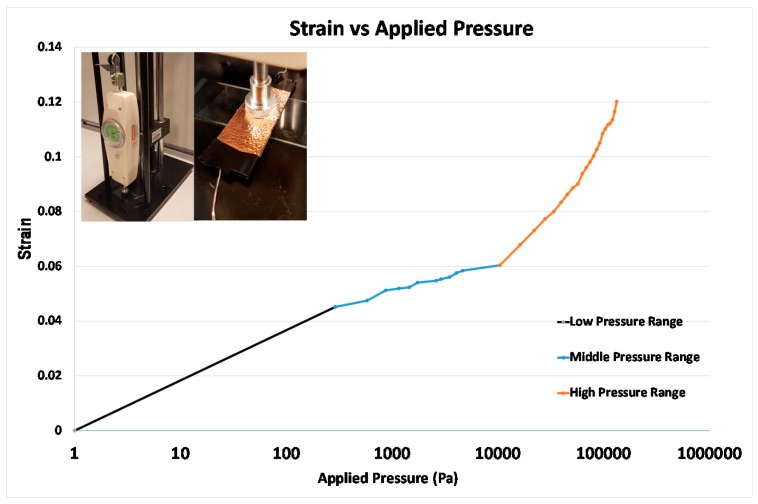
Result of strain change as the pressure is applied from 1 Pa to 131.83 kPa, applied pressure is in logarithmic scale (error bar is too small to be displayed). The pressure is applied through the equipment on the top left side of the graph. The size of the capacitor is 55 mm × 21 mm × 890 µm and initial capacitance is 61.63 pF. The graph is divided into three ranges: low pressure range (0–289 Pa), mid pressure range (289 Pa–10.408 kPa) and high pressure range (10.408–131.83 kPa). These three ranges have different slopes (slope of low pressure range = ~2 × 10^−4^, slope of middle pressure range = ~1 × 10^−6^, and slope of low pressure range = ~4 × 10^−7^).

The strain can be calculated by using the equation, *C* = *ε*_o_*εA*/*d* (where *ε*_o_ is dielectric constant of free space, *ε* relative dielectric constant of the material, A is area of electrode, *d* is the distance between each electrode). The result of measured strain is shown in Figure 8. The graph was obtained by averaging values from five repetitive experiments, and the slope was obtained by drawing a trend line using Microsoft Excel (Microsoft Excel 2013, Redmond, WA, USA). The 1/slope of the graph is Young’s modulus (Young’s modulus = pressure/strain) of dielectric material of the pressure sensor. The dielectric layer of the pressure sensor is composed of three different materials: air, PDMS (15:1) and PDMS (5:1). These materials have a different Young’s modulus, PDMS (15:1) = 1.4 MPa and PDMS (5:1) = 3.59 MPa [34] (air does not have Young’s modulus because it is not solid). Due to the difference in the Young’s modulus of each material, we expect that each material would deform at different applied pressures (three ranges of pressure because there are three materials composing the dielectric layer). The strain change can be divided into three ranges: low pressure range (1–289 Pa), middle pressure range (289 Pa–10.408 kPa), and high pressure range (10.408–131.81 kPa).

Unlike our expectation in designing the pressure sensor, each range seems to correspond to the deformation of the combination of materials. In the low pressure range, the air gap was deformed and its Young’s modulus from the graph is 5000 Pa (the slope is ~2 × 10^−4^). This does not mean that air has Young`s modulus, but, as the air gap was fabricated within the PDMS (15:1), the calculated value would require pressure to deform the air gap with the PDMS (15:1) (the main deformation would be generated by air). In the middle pressure range, the PDMS (15:1) that has lower Young’s modulus was deformed and the calculated Young’s modulus from the graph is ~1 MPa (the slope is ~1 × 10^−6^). The theoretical Young’s modulus of PDMS (15:1) is 1.4 MPa, which is greater than our value. This would be caused by the existence of an air-gap that decreased the Young’s modulus of the middle pressure range (the PDMS (15:1) was mainly deformed). In the high pressure range, PDMS (5:1) was deformed and the calculated Young’s modulus from the graph is ~2.5 MPa (the slope is ~4 × 10^−7^). The theoretical Young’s modulus of PDMS (5:1) is 3.59 MPa, which is greater than our value because the deformation by the high pressure range was done by a combination of the PDMS (15:1) and the PDMS (5:1).

Due to the combined material deformation, further studies for optimization would be required to test in a practical experiment. Moreover, further study is required for the demonstration of a flexible pressure sensor because, when the pressure sensor is bent, the capacitance change is unstable. At this time, we demonstrate a possible application of our method through fabricating flexible pressure sensors.

### 3.6. The Result of Different Metal Transfers

**Figure 9:**
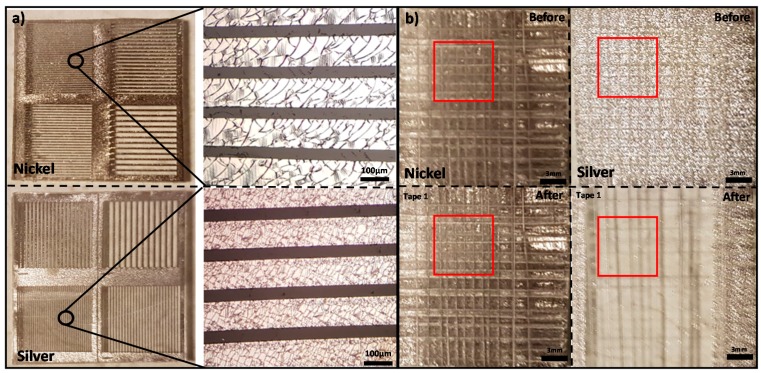
(**a**) Images of transferred micro-size line pattern of nickel and silver. The line width is different, 400 µm, 300 µm, 200 µm and 100 µm, which were transferred onto PDMS. This shows that micro-size of nickel and silver can be transferred on PDMS; (**b**) the result of cross-hatch adhesive test for silver and nickel. The result shows that the adhesion strength of nickel is strong, and the ASTM class is 5B. However, the adhesion strength of silver is weak, and ASTM class is 0B.

The chemical-free metal PDMS thermal bonding is compatible with nickel and silver as shown in Figure 9a. As the result shows, the transfer of a micro-size pattern of nickel and silver from the adhesive layer (chromium) was successful using our method (as well as copper). Additionally, our method can transfer the micro-size pattern on PDMS with the widths of silver and nickel 400 µm, 300 µm, 200 µm and 100 µm easily transferred on PDMS. The possibility of transferring micro-size patterns of nickel allows fabrication of flexible soft magnets and soft magnetic structure integrated microfluidic devices. The soft magnetic structures in microfluidic devices can be used in magnetic field based particle sorting or bioassay [35,36]. Additionally, the possibility of transferring micro-size patterns of silver allows fabrication of sensors as well.

The result of the cross-hatch adhesion test of nickel and silver is shown in Figure 9b. The ASTM class of nickel adhesion to PDMS is 5B because 0% nickel was removed by the tape. The ASTM class of silver adhesion to PDMS is 0B because more than 65% silver was removed by the tape. The result shows that the adhesion strength between nickel and PDMS is strong; however, the adhesion between silver and PDMS is weak. It seems that the adhesion strength between silver and PDMS is not stable (mostly weak but strong adhesion can be generated at low success rate) and transferred silver is easily damaged. This indicates that the silver transfer requires more optimization, which requires further studies.

Similar to cracks on transferred copper, there are cracks on transferred nickel and silver. This is also caused by the thermal expansion mismatch and the same strategy for copper (such as serpentine design) can be studied in the future to avoid cracking.

There are other metals that are compatible with our method such as aluminum, chromium and permalloy. However, the transfer of these metals is difficult because the condition is not optimized. This process is still developing; therefore we believe that further studies of this method can enable the transfer of aluminum, permalloy and other metals.

## 4. Conclusions

In this paper, we introduced and demonstrated a chemical-free metal PDMS thermal bonding, which is the same as the PDMS curing process but with different curing conditions. Since no chemical treatment is required, our process is simpler and faster compared to traditional methods. As a simple application of our method, fabrication of a flexible copper electrode was demonstrated. The study of the optimum thickness of an electrode using copper proved that the optimum thickness of the copper electrode was approximately 500 nm, which was the most resistant to the deformation such as bending.

By applying a serpentine design in the fabrication of electrodes before transfer, the quality of a flexible electrode after transfer can be improved. Moreover, apart from copper, various metals such as nickel and silver are compatible with our method. Thus, we expect that our method can be used in the fabrication of electrodes (sensors as well) or soft-magnetic structure integrated microfluidic devices, such as a microfluidic device with a micro-heater and magnetic particle guiding/sorting utilities.

## Figures and Tables

**Table 1:** The list of various PDMS curing conditions tested for studying the conditions that create a strong bonding.

Trial	Temperature (°C)	Mixing Ratio	Baking Time (min)	Transfer Result
1	100	20:1	20	X
2	100	10:1	20	X
3	100	5:1	20	X
4	100	5:1	30	Δ (~4%)
5	100	5:1	45	Δ (~6%)
6	150	20:1	20	X
7	150	10:1	20	X
8	150	5:1	20	O
9	150	5:1	30	O
10	150	5:1	45	O

X: No transfer; Δ: Partial Transfer; O: Full Transfer (works with copper, nickel and silver).

**Table 2:** The sheet resistance of copper (thickness from 75 nm, 150 nm, 300 nm, 500 nm and 1000 nm) measured before and after transfer.

Copper Thickness	Sheet Resistance before Transfer	Sheet Resistance after Transfer	Sheet Resistance Increase Rate
75 nm	0.446 ± 0.004 Ω/sq	1.22 ± 0.06 Ω/sq	63.43%
150 nm	0.199 ± 0.003 Ω/sq	0.22 ± 0.006 Ω/sq	9.57%
300 nm	0.0876 ± 0.002 Ω/sq	0.0791 ± 0.003 Ω/sq	−10.69%
500 nm	0.053 ± 0.001 Ω/sq	0.045 ± 0.0008 Ω/sq	−17.1%
1000 nm	0.0121 ± 0.002 Ω/sq	0.0108 ± 0.0006 Ω/sq	−12.35%
